# Utilization of youth corners: a model for improving youth-friendly health services in Neno District, Southern Malawi

**DOI:** 10.1080/16549716.2025.2586278

**Published:** 2025-11-17

**Authors:** Timothy Kavuma, Stellar Chibvunde, Vera Kabanda, Fabien Munyaneza, Kondwani Mpinga, Isaac Mphande, Basimenye Nhlema, Christopher Banda, Jimmy Harare, Enoch Ndarama, Antony Sandiyang’ane, Moses Banda Aron

**Affiliations:** a Informatics Department, Partners in Health Abwenzi Pa Za Umoyo, Neno, Malawi; b Health System Strengthening, Global Health Corps, Lilongwe, Malawi; c Ministry of Health, Neno District Health Office, Neno, Malawi; d Research Group Snakebite Envenoming, Bernhard Nocht Institute for Tropical Medicine, Hamburg, Germany

**Keywords:** adolescents’ health, sexual reproductive health, youth corners, youth friendly health service, health care utilization, health care access

## Abstract

**Background:**

Adolescents in low- and middle-income countries often struggle to access sexual and reproductive health services (SRHS). To address this, the Ministry of Health, with support from Partners In Health, implemented Youth Corners in selected health facilities in Neno District to improve the utilisation of Youth-Friendly Health Services (YFHS).

**Objective:**

This study examines the role of Youth Corners in enhancing YFHS usage in Neno District, Malawi.

**Methods:**

A descriptive cross-sectional study was conducted by extracting data from Youth Corner registers and DHIS2 from health facilities between October 2022 and September 2023. Population estimates were obtained from the National Statistical Office projections. Descriptive statistics and chi-square tests were used to describe service utilization patterns, and binary logistic regression was used to assess the association between distance and service attendance.

**Results:**

During the study period, 1,877 young people aged 10–24 accessed YFHS, with 45% (*n* = 852) aged 15–19 and 57% (*n* = 1,063) female. They completed 2,869 visits, all receiving information and counselling. The utilisation rate was 829 visits per 1,000 young people aged 10–24. An association was found between distance, age, and attendance (*p* < 0.05). Adolescents aged 15–19 were less likely to attend youth corners (OR = 0.57, *p* = 0.003), and those living more than 10 km from facilities participated less frequently (OR = 0.59, *p* = 0.011).

**Conclusion:**

While Youth Corners are highly suggested as a way for improving YFHS uptake among young people, their impact in Neno District was found to be suboptimal.

## PAPER CONTEXT

- **Main findings:** We found the sub-optimal role of Youth Corners influencing Youth Friendly Health Services uptake in Neno district and that distance and age significantly influence adolescents’ access to Youth Corners influencing Youth Friendly Health Services, with those aged 15–19 and those living more than 10 km from health facilities being less likely to attend, highlighting critical barriers to healthcare utilization among adolescents.
- **Added knowledge:** The finding that adolescents aged 15–19 were less likely to attend Youth Corners influencing Youth Friendly Health Services than other age groups (despite being in a high-risk category for many health issues) adds to the understanding of age-related barriers in healthcare utilization. This suggests a gap in engagement for older adolescents, calling for targeted interventions that consider the unique challenges faced by this group.
- **Global health impact for policy and action:** The study highlights that distance to healthcare facilities significantly affects adolescents’ access to Youth Corners influencing Youth Friendly Health Services, with those living more than 10 km away attending less frequently. This finding underscores the need for global health policies that prioritize geographical accessibility in the design of adolescent health programs. Governments and health organizations should consider expanding services through mobile health units, community health workers, or telemedicine to reach adolescents in remote areas.

## Background

The World Health Organization defines ‘young people’ as individuals aged 10–24 years, with those aged 10–19 years referred to as ‘adolescents’ and those aged 15–24 years as ‘youth.’ [1]. Young people constitute 24% of the total global population, estimated at 1.8 billion, of which 90% live in low‐middle income countries (LMICs) [1–4]. In East and Southern Africa, they comprise 32% of the population, while in Malawi, this figure is slightly higher at 35% [4]. Young people in many LMICs face numerous challenges, including limited access to education and healthcare, which have long-term impacts on their lives [2,5,6].

While access to healthcare remains a challenge in many LMICs, in rural areas, factors like cultural norms and perceptions [7], quality of services [8,9], distance to health facilities and travel time make access to health facilities more costly [10]. Compared to the general population, adolescents are disproportionately affected in accessing healthcare [2,11–14]. Literature suggests that limited access to sexual and reproductive health and rights (SRHR) among adolescents leads to various adverse health outcomes, including early pregnancy and high rates of Sexually Transmitted Infections (STIs) [14–16]. Studies have also suggested that the available services are often not tailored to the needs of young people, resulting in low access and utilisation rates [12,13,17]. Health services should be of good quality, defined as the best possible care provided to the patient [18]. However, in many LMICs, the low quality of services and the limited investment in youth-friendly health services are particularly evident as governments are prioritising competing demands such as child health, emerging epidemics, and climate change [8,19]. And yet, the impact of lost productive hours due to illness or poor health, particularly among young people, affects not only their well-being but also their families and future [20].

Malawi, a low and middle-income country in southern Africa, is not exempted from the disparities young people face in access to health. To address these challenges, in 2007, the Malawi government launched the Youth-Friendly Health Services National Standards and the Youth-Friendly Health Services (YFHS) program [21]. YFHS are designed to be relevant, accessible, attractive, affordable, appropriate, and acceptable to young people [21]. In 2022, the government developed the Malawi National Youth Policy 2023–2028, which promotes strategies and programs that i) youth have access to quality YFHS that are safe, respect their right to privacy, confidential, and affordable while respecting their cultural values and religious beliefs, ii) promote access to HIV and STI prevention and treatment, and family planning services for youth with particular attention to adolescent girls and young women, iii) advocate for improved provision of and access to quality and integrated YFHS with a deliberate focus on rural populations, and iv) support capacity building of health service providers, stakeholders, youth, and youth-led organisations in delivering YFHS [22]. Assessing the uptake of services that contribute to achieving program objectives is essential. For example, in YFHS, uptake would be defined as the proportion of young people accessing these services.

In 2018, a study done in Malawi found that youth were more likely to access health care in YFHS than in standard care delivery [23]. However, ample literature suggests that many youth still have limited access to YFHS [12,14,24,25]. Social-economic, cultural, and religious barriers still affect access and utilization of YFHS among young people in Malawi [11,12,24], coupled with low investment in YFHS [19]. Furthermore, personal factors, including gender, age, awareness, and structural factors such as availability of printed health education materials, provider attitudes, and fear or shyness about seeking services, all contribute to low utilization of YFHS [24]. Integrating these services into private clinics is costly and cannot be accessed by most Malawian youth [26]. Moreover, while urban areas typically have better access to YFHS, young people in rural areas are disadvantaged due to limited awareness of the availability of these services [7,27]. This calls for innovative ideas to ensure everyone has access, for example, in an appropriate way, such as ‘youth corners’ [7,27–29]. Youth corners are separate spaces for young people within health facilities in which adolescent and youth-friendly SRH information and services are provided [30]. Youth Corners are considered a primary significant driver with the potential to enhance SRH outcomes and address challenges such as low contraceptive uptake and high teenage pregnancy rates among young people [24].

Partners in Health (PIH), also known as Abwenzi Pa Za Umoyo (APZU) in Malawi, has accompanied the Ministry of Health since 2007 to support healthcare services in the country [31]. PIH focuses on implementing comprehensive clinical and community outreach programs to improve healthcare services. In the Neno district, they have supported 14 health facilities, successfully implementing the Youth Corner model to provide health services to young people. However, no previous studies have described the uptake of YFHS in the Neno district or assessed the role of Youth Corners in improving YFHS.

## Objectives

The researchers aimed at providing a comprehensive analysis of the utilization and uptake of YFHS in four designated health facilities with Youth Corners (YFHS-facilities). Further, they compared the access to YFHS between YFHS facilities with 10 other facilities without Youth Corners (Non-YFHS facilities). Finally, to identify barriers affecting YFHS uptake, the researchers assessed the impact of the distance between the participants’ villages and visited health facilities on their attendance at Youth Corners.

## Methods

### Study design and setting

**Figure 1.**
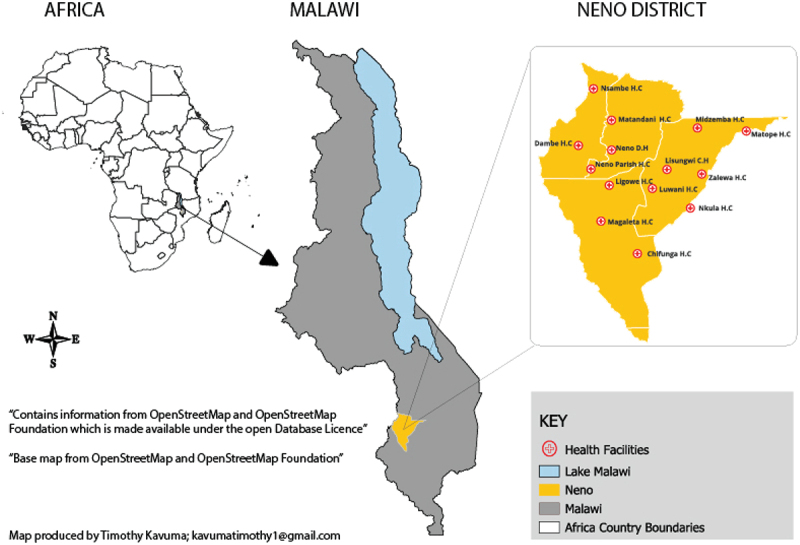
Map of Neno district health facilities.

The researchers conducted a descriptive cross-sectional study in the Neno district. Neno is in the Southern region of Malawi and shares a border with Mozambique to the west. The district‘s landscape makes it a hard-to-reach area with poor roads. As of 2023, Neno district had an estimated population of 153,132 people, of which 51,191 were young people aged 10–24 [22]. Most people in Neno depend on rainfed, unindustrialized agriculture as their primary source of household income. The district is served by 15 health facilities, including 13 primary care levels and two hospitals, all operated by and overseen by the Ministry of Health (MOH), with key support from Partners in Health (PIH) and other implementing partners (Figure 1).

### Program setting

In 2019, PIH, with funding from Global Affairs Canada in collaboration with the Ministry of Health, launched the ‘No Woman or Girl Left Behind’ project in Neno District health office, aiming to improve the provision of gender-sensitive and rights-based sexual and reproductive health services. Youth corners were part of this project’s programming and were gradually rolled out with keen emphasis on having dedicated spaces where youth can interact, discuss and access services.

#### Space identification

Suitable spaces where both SRHR services and recreational activities were coordinated. Matope and Nsambe had dedicated spaces for Youth Corners, while Dambe and Nsambe utilized out-patient department spaces in the afternoon.

#### Needs assessment

Young People’s preferences for recreational activities and SRHR were used to understand the youths’ expectations and how they envisioned SRHR service provision in youth corners.

#### Recreation materials provision

The necessary games and equipment needed to set up and run recreational activities were provided. This ensured that participants had access to engaging and enjoyable resources in addition to the SRHR services that they intended to access.

#### Training

Service providers and other support staff who manage the youth corners and activities were trained utilizing the YFHS manual.

The program was designed to provide high-quality YFHS with added components leading to Income-generating Activities and Edutainment, this is because Edutainment is a key component in Youth mobilization and retention [15]. YFHS provided in Youth Corners include: Information & counselling, Family planning services, Sexual Tramissimited Infection (STI) management, HIV testing services (HTS), Antiretroviral Therapy (ART), Post-Exposure Prophylaxis (PEP), Pre-Exposure Prophylaxis (PrEP), Sexual and gender-based violence (SGBV), Condoms provision, Drug and substance use on top of access to other health care services. Youth Corner registers were introduced in October 2022 to migrate from the traditional hardcover attendance sheets. Each facility organized youth corners twice a month. From the onset of 2023, a youth corner was organized each week for each facility: Wednesdays for Chifunga and Matope Health Centres, and Mondays for Dambe Health Centre and Nsambe Health Centre. YFHS data gathered through Youth Corners is compiled together with data from other programs to produce a monthly YFHS facility report.

### Target population

The study population comprised young people who sought YFHS from the study facilities in Neno District. For family planning, we focused on two age categories: 15–19 and 20–24. This population was selected to encompass the range of definitions present within scientific literature. The other age categories were, however, maintained to describe the role of Youth Corners at health facilities; they were also used to differentiate the impact distance has on service utilization among different age groups.

### Sampling techniques

The researchers performed a retrospective review of existing data from Youth Corner registers and sourced comparative data from DHIS2, specifically from facilities’ Youth Friendly Health Services monthly reports for the study period. Researchers excluded records without visit dates and patient age and included data with complete records, i.e. date, name, age, and service provided.

### Data collection tools and techniques

We developed a CommCare application based on the variables from the registers. We then hired and trained two data collectors to abstract the data. Between 1 and 28 February 2024, the data collectors extracted information from the registers in the selected Youth Corner facilities: Nsambe HC, Dambe HC, Chifunga HC, and Matope HC for our study period. We further extracted monthly aggregated level data from the DHIS2 system on YFHS other than the Youth Corner registers by youths aged 10–24. Further, we sourced the adolescent population projections for the catchment area from the 2018–2020 projection by the Malawi National Statistics Office [22]. During the study period, the Youth Corner model catchment areas’ 10–24 years adolescent population was projected to be approximately 19,162 (Chifunga: 3817, Dambe: 4800, Matope: 5660, and Nsambe: 4885). Finally, we used Google Maps to calculate the distance in kilometres between villages and facilities along demarcated roads. The data were captured in an Excel sheet and later merged with the data from Commcare and DHIS2 for analysis.

### Data processing and analysis

We exported data from CommCare to Excel, cleaned it using Python, and later used R version 4.4.0 for analysis. We categorized age and distance and computed descriptive statistics to characterize the youths, including their demographics and the services they utilized during the study period. Youth Corner attendance was presented as frequency to compare individual facility youth corner attendance. To further assess the role of youth corners, the monthly facility attendance was divided by the catchment area’s population to produce the percentage of the adolescent population served by the youth corners during the study period. We used bar charts to compare the visits per 1,000 population of those aged 10–24 years to measure the utilization of YFHS across the 14 supported facilities in Neno, which was also used to assess the role of Youth Corners on the promotion of YFHS utilization. Using Chi-square and binary logistic regression, we evaluated the association between distance, age and frequency of visits.

## Results

### Demographic characteristics of participants (N = 1,877)

During the study period, 1,877 young people were registered to have accessed youth corners at the four facilities. Over half (n = 1063, 57%) of the attendees were female. Nearly half (n = 852, 45%) of all attendees were within the 15–19 age group, while those aged 25+ years accounted for only 7.9% of the total. Of these, 57% were female. Attendees were fairly distributed across the four facilities, with Matope and Dambe Health Centres having the highest attendance (n = 598, 31.9%).

### Youth Corners attendance

**Figure 2.**
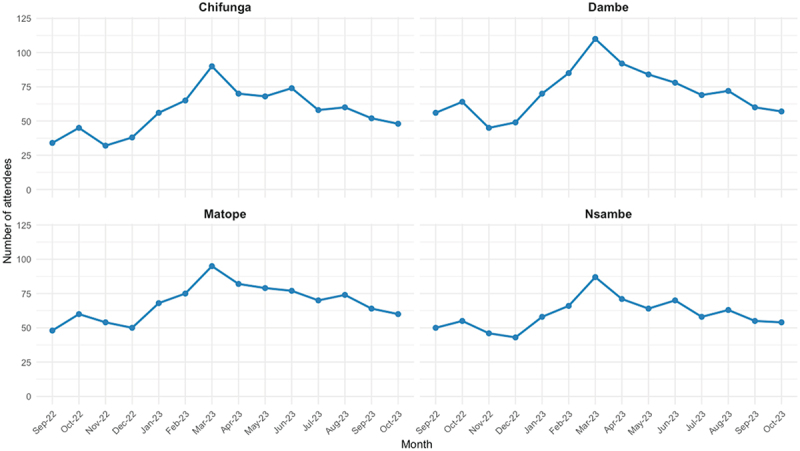
Youth attendance by month and facility.

Across all four facilities, March 2023 had the highest Youth Corner attendance, followed by May 2023, while November 2022 and December 2022 had the lowest attendance (Table 1, Figure 2).

**Table 1.** Demographic characteristics of Youth accessing Youth Corners October 2022–September 2023.

	Health facility				
Variables n (%)	Chifunga, *N* = 260	Dambe, *N* = 598	Matope, *N* = 598	Nsambe, *N* = 421	Overall, *N* = 1,877
**Age Category**					
10–14	65 (25%)	11 (1.8%)	115 (19%)	31 (7.4%)	222 (12%)
15–19	68 (26%)	296 (49%)	241 (40%)	247 (59%)	852 (45%)
20–24	89 (34%)	278 (46%)	170 (28%)	117 (28%)	654 (35%)
25+	38 (15%)	13 (2.2%)	72 (12%)	26 (6.2%)	149 (7.9%)
**Gender**					
Female	129 (49%)	371 (62%)	319 (53%)	245 (58%)	1,063 (57%)
Male	132 (51%)	227 (38%)	279 (42%)	176 (42%)	814 (43%)

Regardless of age group, Matope Health Centre had 60% of the overall March monthly attendance, which was also associated with the rigorous community mobilization exercise that preceded the event. Chifunga generally performed lower than the other facilities.

**Figure 3.**
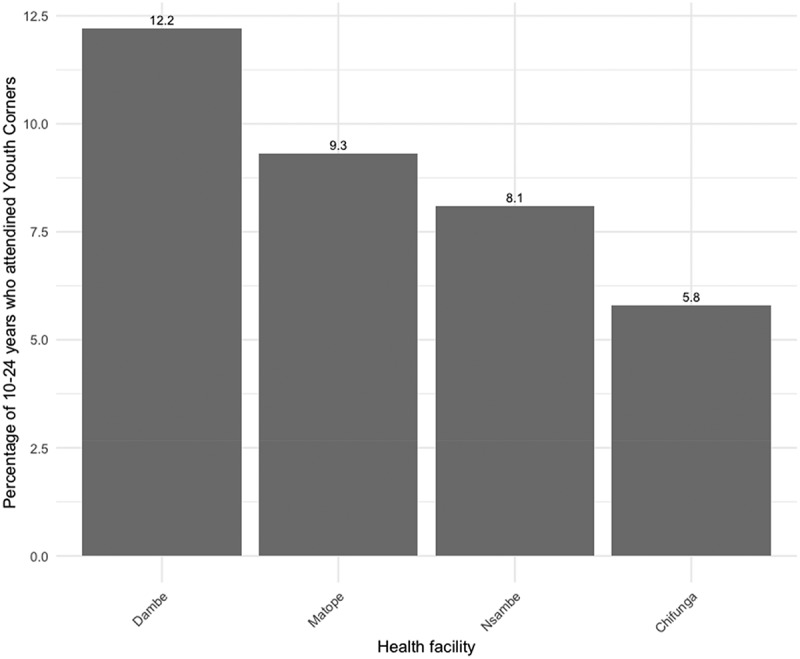
Percentage of 10–24 youth who attended youth corners at selected facilities.

Dambe Health Centre recorded the highest percentage (12.2%) of 10–24-year-old adolescents within the catchment area who attended Youth Corners. This was followed by Matope Health Centre, with 9.3%, while Chifunga Health Centre had the lowest percentage of the population served (Figure 3).

### Youth Corner service utilization patterns

Out of the 3,024 completed visits, everyone accessed information and counselling in addition to other services available through the program each time they attended the scheduled Youth Corner. Therefore, this was considered a service delivered for each visit, making the utilization rate 100% (*n* = 3,024), followed by the HIV Testing Service (15%, *n* = 470), and Condom provision (12%, *n* = 377).

Apart from PEP and Information and counselling, Dambe Health Centre had the highest number of visits where other services were provided; ART (*n* = 6, 75%), Condom Provision (*n* = 246, 65%), Drug and Substance Abuse (*n* = 4, 50%), Family Planning (*n* = 107, 50%), HTS (*n* = 381, 81%), STI Management (*n* = 44), SGBV (*n* = 1, 50%) (S1 Table).

### Impact of distance

Researchers found a significant association between the distance travelled by Youth Corner attendees, the age of individuals, and the likelihood of repeat visits. Participants within a distance greater than 10 km from the Facility and age groups (15–19 years and 20–24 years) categories were independently associated with lower odds of repeat visits. These associations were generally strengthened when adjusting for other factors in the multivariable analysis (Table 2).

**Table 2.** Relationship between distance, age and subsequent attendance.

	Bivariable Binary regression		Multivariable Binary regression	
Covariate	OR (95%CI)	*p*-value	OR (95%CI)	*p*-value
**Distance category**				
<5	Reference	Reference	Reference	Reference
5–10	0.82(0.61, 1.10)	0.200	0.83 (0.61, 0.91)	0.200
10+	0.59(0.39, 0.87)	**0.011**	0.61 (0.40, 0.91)	**0.020**
**Age group**				
10–14	Reference	Reference	Reference	Reference
15–19	0.57(0.39, 0.83)	**0.003**	0.60(0.42, 0.88)	**0.008**
20–24	0.67(0.46, 0.98)	**0.037**	0.73(0.50, 1.09)	0.120
25+	1.39(0.86, 2.23)	0.200	1.49(0.92, 2.41)	0.110

1 OR = Odds Ratio, CI = Confidence Interval.

### Comparison of facility visits per 1,000 population

**Figure 4.**
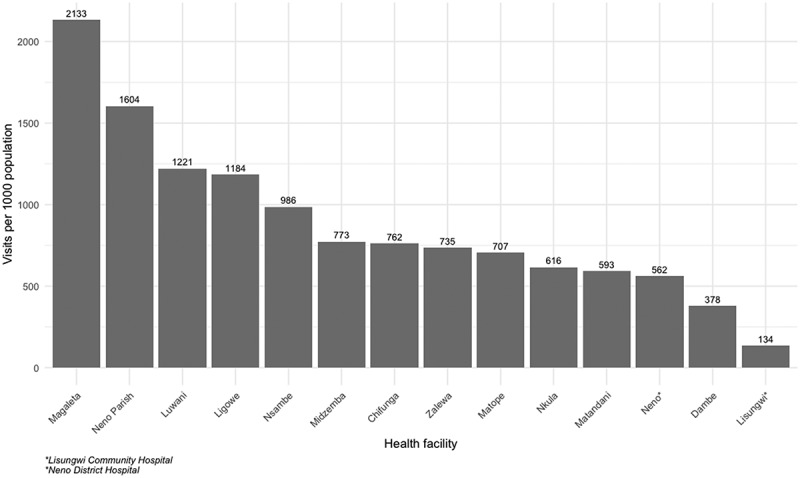
10–24 year YFHS visits per 1,000 population.

On the spectrum of visits per 1,000 population, the top three facilities were non-Youth Corner facilities (Magaleta Health Centre, Neno Parish Health Centre and Luwani Health Centre), and the least three were Lisungwi Community Hospital, Dambe Health Centre and Neno District Hospital (Figure 4).

## Discussion

To the researchers’ knowledge, there has not been any study conducted to describe the role of the Youth Corners in promoting access and uptake of YFHS in rural Malawi. This study found that the Youth Corners model provided a platform for increased access to YFHS in Neno. Indicators in this study, such as frequency of visits, proportion of 10–24 youth Corner attendance, reveal that utilization of YFHS in Neno remains low; this could be associated with factors that have been highlighted by prior studies in the area, such as provider attitudes, religious beliefs, and public perceptions as barriers to YFHS uptake [12,24,29,32]. The researchers’ findings reinforce findings documented in past literature, indicating underutilization of YFHS in Malawi [24,33,34].

During the study period, Youth Corners facilitated YFHS access to 1,877 young people, the majority aged 15–19 years (45% of the participants) and predominantly female. This demographic profile reflects the targeted age group for YFHS. For the same age group, female representation (57%) was slightly higher than male adolescents. These findings were consistent with a study conducted in Malawi and Tanzania in 2016, which revealed that males are also equally involved in family planning service utilization in Malawi [34], which is deduced by relative female and male youth corner attendance. This further shows that the provision of YFHS is equally beneficial and relevant for both male and female young people.

Researchers found that despite equal demand for Youth Corners by male and female participants, the services accessed varied according to gender. In addition to Information and Counselling, which was accessed by each attendee, more female attendees accessed HIV testing services, family planning, STI management, and ART services compared to male adolescents. Male attendees, however, accessed Condoms and PEP services more than female attendees.

Further, researchers found a significant relationship between age and Youth Corner attendance, with those aged between 15 and 19 less likely to attend more frequently (OR = 0.57, *p* = 0.003). Similar findings were reported by a study conducted in Lilongwe, the capital of Malawi, which highlighted that incentives used to attract female adolescents to seek YFHS; these included paying for their transport, operating youth-friendly clinics at suitable hours [35]. The same literature highlighted barriers such as negative provider attitudes and fear of confidentiality. Eliminating such barriers is a crucial step in improving the uptake of YFHS, this is because most recent literature still flags the same issues as highlighted by past literature [2,12,35,36].

Prior studies have highlighted that the provision of YFHS in dedicated spaces within health facilities increases comfort levels among young people, thereby encouraging regular visits for various health needs, including family planning, STI management, and HIV testing services [2,3,12,29,35]. However, this study reveals that the space alone is insufficient to promote YFHS uptake. The best-performing facility during the study period (Dambe Health Centre) did not have a designated space for Youth Corner schedules. Youth Corners were conducted utilizing OPD facilities in the afternoons on Youth Corner days. Researchers also observed that, on top of this facility having the highest population proportion reached through Youth Corners, the facility provided the highest number of services other than information and Counselling. The two facilities recorded to have dedicated Youth Corner spaces (Matope Health Centre and Nsambe Health Centre), ranked 2nd and 3rd, respectively, in the population proportion reached. This highlighted that on top of dedicated spaces, there are other qualitative factors influencing Youth Corner attendance, such as providers’ attitudes stated in other studies, which were out of this study’s scope [13,24,35].

Our study revealed low Youth Corners attendance and low uptake of YFHS in Neno, only 10% of 10–24-year-old adolescents accessed Youth Corners to seek YFHS during the study period. A study conducted in Blantyre, a border district to Neno, indicated less than half of the participants (43%) had ever accessed YFHS, the study highlighted age, gender, knowledge, signage, printed health education materials, provider attitudes and participants being shy or afraid of being seen at the services as barriers to the uptake of YFHS [24], this underscores is reinforced through the researchers findings of age being a general factor for the low utilization of YFHS in Neno on top of other specific challenges such as distance.

Distance emerged as a significant factor affecting the utilization of YFHS in Neno District. Adolescents residing more than 10 kilometres from health facilities were less likely to access Youth Corners regularly. This finding aligns with previous research emphasizing the correlation between travel distance and healthcare utilization, particularly in rural settings [1,4]. Future interventions should consider strategies to mitigate the impact of distance, such as mobile Youth Corners and mobile health units or outreach programs targeting remote communities, as suggested by prior studies [37].

The study revealed variations in 10–24 years’ YFHS visits per 1,000 population between facilities running the Youth Corner model and those offering YFHS through OPD with an average of 829 visits. In addition to utilization, this indicator directly represents OPD utilization which measures the availability and quality of services. People are more likely to attend facilities when barriers to entry (cost, distance etc.) and when they feel that they receive quality services. Furthermore, this indicator measures the patient load in a health facility and can be used for planning [38]. Prior studies in the country show that facility visits are highly influenced by the quality of services provided and health status [24,39,40].

This study reveals variations in service utilization, with spikes in attendance in some months. Months with high Youth Corner attendance were attributed to community mobilization and engagement. Prior literature conducted in Malawi found that service utilization increased with multimedia and community motivation [41]. The difference in service utilization across facilities was further solidified through the variation in visits per 1,000 10–24-years population. This variation indicates differences in the quality of services provided at the different facilities.

Several limitations should be considered when interpreting our findings. The data collection relied on facility records and may not capture all factors influencing YFHS utilization, such as individual behaviours and perceptions. Additionally, the data available could not provide some equity considerations, such as out-of-school youth, adolescents with disabilities, and marital status. The inclusion criteria were complete records from facility registers with 10+ participants of age; due to incomplete records, approximately 5% of visits could not be considered for this study.

## Conclusion

While Youth Corners in Neno District provide an opportunity to enhance the uptake of YFHS among young people by providing a platform that focuses on young people’s SRHR needs, its utilization remains suboptimal. By addressing barriers related to accessibility and socio-economic factors, these dedicated spaces play a crucial role in improving SRHR service outcomes. In addition to addressing the barriers, mobile outreach clinics and school-based youth corners should be considered to increase accessibility of YFHS, especially for young people who are currently disadvantaged by long distances. Future research should focus on longitudinal studies and qualitative assessments to further explore the sustained impact of Youth Corners on community health and well-being, specifically in remote areas such as Neno.

## Supplementary Material

S1 Table YFHS utilization by sex_age_and_health facility.docx
