# Supplementary material for: Utilization of youth corners: a model for improving youth-friendly health services in Neno District, Southern Malawi
Source: Glob Health Action. 2025 Nov 17;18(1):2586278. doi: 10.1080/16549716.2025.2586278 (PMC12624900; doi:10.1080/16549716.2025.2586278)
Supplement: S1 Table YFHS utilization by sex_age_and_health facility.docx [file ZGHA_A_2586278_SM2220.docx]

**YFHS utilization by sex, age and health facility**

| Service Provided | Sex | | Age group | | | | Health facility | | | |
| --- | --- | --- | --- | --- | --- | --- | --- | --- | --- | --- |
|  | Female  N = 2288 | Male  N = 1715 | 10-14  N = 340 | 15-19  N = 1734 | 20-24  N = 1557 | 25+  N = 372 | Chifunga  N = 602 | Dambe  N = 1417 | Matope  N = 1259 | Nsambe  N = 725 |
| Information and Counseling | 1593 (70%) | 1276 (74%) | 315 (93%) | 1188 (69%) | 1047 (67%) | 319 (86%) | 570 (95%) | 629 (44%) | 1146 (91%) | 520 (72%) |
| ART^1^ | 6 (0.3%) | 2 (0.1%) | 0 (0%) | 3 (0.2%) | 5 (0.3%) | 0 (0%) | 0 (0%) | 6 (0.4%) | 1 (<0.1%) | 1 (0.1%) |
| Condom Provision | 167 (7.3%) | 209 (12%) | 11 (3.2%) | 180 (10%) | 165 (11%) | 20 (5.4%) | 14 (2.3%) | 246 (17%) | 77 (6.1%) | 39 (5.4%) |
| Drug and Substance use | 4 (0.2%) | 4 (0.2%) | 0 (0%) | 2 (0.1%) | 6 (0.4%) | 0 (0%) | 0 (0%) | 4 (0.3%) | 2 (0.1%) | 2 (0.3%) |
| Family Planning | 184 (8.0%) | 31 (1.8%) | 4 (1.2%) | 97 (5.6%) | 99 (6.4%) | 15 (4.0%) | 7 (1.2%) | 107 (7.6%) | 23 (1.8%) | 78 (11%) |
| HTS^2^ | 301 (13%) | 169 (9.9%) | 8 (2.4%) | 237 (14%) | 209 (13%) | 16 (4.3%) | 6 (1.0%) | 380 (27%) | 2 (0.2%) | 82 (11%) |
| PEP^3^ | 2 (<0.1%) | 3 (0.2%) | 0 (0%) | 1 (<0.1%) | 4 (0.3%) | 0 (0%) | 1 (0.2%) | 0 (0%) | 3 (0.2%) | 1 (0.1%) |
| SGBV^4^ | 1 (<0.1%) | 1 (<0.1%) | 1 (0.3%) | 1 (<0.1%) | 0 (0%) | 0 (0%) | 0 (0%) | 1 (<0.1%) | 1 (<0.1%) | 0 (0%) |
| STI Management^5^ | 30 (1.3%) | 20 (1.2%) | 1 (0.3%) | 25 (1.4%) | 22 (1.4%) | 2 (0.5%) | 0 (0%) | 44 (3.1%) | 4 (0.3%) | 2 (0.3%) |
| ^1^Antiretroviral therapy; ^2^HIV testing services; ^3^Pre Exposure Prophylaxis; ^4^Sexual Gender Based Violence; ^5^Sexual transmission infection | | | | | | | | | | |
